# Unraveling the Effects of Climate Change and Human Activity on Potential Habitat Range Shifts in Four *Symplocos* Species in China

**DOI:** 10.3390/plants14203200

**Published:** 2025-10-18

**Authors:** Zongfeng Li, Yuhong Sun, Wenke Chen, Chengxiang Sun, Wenjing Tao, Jianping Tao, Weixue Luo, Jinchun Liu

**Affiliations:** 1 Key Laboratory of Eco-Environments in Three Gorges Reservoir Region (Ministry of Education), Chongqing Key Laboratory of Plant Ecology and Resources Research in Three Gorges Reservoir Region, School of Life Sciences, Southwest University, Chongqing 400715, China; lizfswu@swu.edu.cn (Z.L.); sunyuh2022@163.com (Y.S.); chenwenke2024@163.com (W.C.); taojp@swu.edu.cn (J.T.); 2 Chongqing Jinfo Mountain Karst Ecosystem National Observation and Research Station, Southwest University, Chongqing 400715, China

**Keywords:** *Symplocos setchuensis*, Maxent model, climate change, anthropogenic influence, potential geographical distribution

## Abstract

Climate change and human activities profoundly impact forest biodiversity, with effects projected to intensify. The *Symplocos* genus, a diverse assemblage of flowering plants prevalent in the subtropical and tropical forests of the Yangtze River in China, holds substantial economic and medicinal value. However, the impacts of climate change and human activities on the habitat ranges of *Symplocos* species in China remain unclear. This study employed an optimized Maxent model to predict potential habitats for four key *Symplocos* species—*Symplocos setchuensis*, *Symplocos chinensis*, *Symplocos groffii*, and *Symplocos sumuntia* under current and multiple future climate scenarios (SSP1-2.6 and SSP5-8.5 during the 2070s and 2090s). Moreover, we assessed the relative importance of various predictors, including climatic, topographic, soil, and anthropogenic factors, in shaping their habitat range patterns. Currently, the habitat ranges of the four *Symplocos* species are mainly concentrated in southern China, exhibiting notable differences in areas of high habitat suitability. Furthermore, the habitat ranges of *S. setchuensis*, *S. chinensis*, *S. groffii*, and *S. sumuntia* were primarily influenced by the mean temperature of the driest quarter (bio9), the minimum temperature of the coldest month (bio6), the temperature annual range (bio7), and precipitation seasonality (bio15), respectively. Notably, the habitat suitability of *S. setchuensis*, and *S. sumuntia* increased at a progressively slower rate with human footprint. Under future climate scenarios, *S. groffii* and *S. sumuntia* are projected to expand their ranges significantly northward, while *S. chinensis* is expected to maintain stable habitat, and *S. setchuensis* may face considerable contractions. Our results underscore the importance of climate and human activities in shaping the habitat ranges of *Symplocos* species, revealing distinct adaptive responses among the four species under future climate change.

## 1. Introduction

*Symplocos* Jacq., the largest genus within the *Symplocaceae* family, encompasses a diverse array of shrubs and trees, predominantly found in tropical and subtropical forests [1,2]. In China, approximately 80 *Symplocos* species are concentrated in the southern regions of the Yangtze River [3,4]. These species are crucial in maintaining biodiversity, retaining water and soil, and providing valuable medicinal resources [5]. Among them, *S. setchuensis*, *S. chinensis*, *S. groffii*, and *S. sumuntia* are the most common species. Specifically, *S. setchuensis*, a small evergreen tree, is widely distributed in southern China and is recognized for its medicinal applications in treating edema, asthma, and as an anti-AIDS agent [6]. *S. chinensis*, a shrub typically found at altitudes of 230–1000 m in southern China, has long been used in traditional medicine to treat colds, fevers, and malaria [7]. Moreover, *S. groffii*, a small tree or shrub, thrives in humid environments and dense forests in southern China and is commonly used in crafting furniture and artisanal items. Finally, *S. sumuntia*, a tree found in mountainous regions at altitudes between 200 and 1500 m in southern China, is used medicinally for treating coughs, tonsillitis, and stomachaches [8]. In recent years, despite numerous studies focusing on the chemistry and pharmacology of the *Symplocos* genus [9], the habitat suitability and ranges of the four *Symplocos* species in China remain poorly understood.

Over the past few decades, climate change has emerged as a major global challenge, exerting immense pressure on local environments and global ecosystems. Research indicates that as the climate warms, isotherms shift poleward and upward to cooler latitudes and higher elevations across the world [10]. Plants are susceptible to climatic changes, and this projected warming trend may escalate the risk of plant species extinction, thereby severely threatening the stability and diversity of global terrestrial ecosystems [11,12]. On the other hand, the habitat distributions of various species may respond differently to climate change, including range contractions, expansions, or remaining stable, posing significant challenges in predicting their adaptation strategies [13,14]. As a result, it is crucial to assess the potential habitat ranges of *Symplocos* species under future climate scenarios to ensure their biodiversity conservation and germplasm resources.

Meanwhile, human activities are another type of key driver that can have both positive and negative impacts on species distribution patterns [15,16]. Over the past decades, the exponential growth in human population size and resource consumption has led to dramatic changes in the global ecological landscape [17]. These changes not only affect local biodiversity but also have far-reaching consequences for species’ habitat ranges [18,19]. Specifically, intensified land use, habitat loss, and other anthropogenic influences have resulted in the extinction of numerous species [20,21]. However, some native species have shown resilience, expanding their habitat ranges in response to human-induced environmental changes [22,23,24]. Moreover, anthropogenic efforts to actively conserve and promote certain tree species for cultural or religious imperatives have played a role in shaping their habitat distributions. In East Asia, for instance, large-scale deforestation and agricultural expansion have historically reduced the habitats of many forest species [25], while ecological restoration programs such as China’s Grain for Green project have facilitated vegetation recovery and species range expansion in degraded landscapes [26,27]. In addition, the long-term protection and cultivation of culturally important trees around temples and villages illustrate how traditional practices can directly shape distributional patterns [28,29]. Therefore, investigating these multifaceted human influences provides a more balanced and regionally grounded perspective on the habitat distribution of *Symplocos* species, which is crucial for developing effective conservation and management strategies.

Species distribution models (SDMs) are essential tools in ecology and biogeography, enabling the evaluation of species habitat suitability based on environmental factors [30,31,32,33]. By integrating species occurrence records and site characteristics, SDMs can predict species habitat suitability across various spatial scales [34]. A variety of SDMs, including Maxent [35], Bioclim [36], Domain [37], GAM [38], GLM [39], and Biomapper [40], have been widely applied in predicting the habitat distributions of plants and insects, as well as in assessing the effects of climate and human activities on species distributions [41,42]. These models are also crucial in assessing the risks of species invasions [43,44]. Among these, Maxent has emerged as a preferred tool due to its robustness with small sample sizes, operational efficiency, precision, and ability to process data rapidly [35,45]. Furthermore, this model has been extensively utilized to project the potential species distribution under future climate scenarios, including for endangered species [46], and medicine plants [47,48]. However, despite its broad application, the predictions of Maxent for the habitat ranges of different *Symplocos* species remain unclear.

In this study, we evaluated the potential impacts of climate change and human activity on the habitat ranges of the four *Symplocos* species (*S. setchuensis*, *S. chinensis*, *S. groffii*, and *S. sumuntia*) and identified the key factors influencing their habitat suitability. Using the optimized Maxent model, we simulated the potential suitable habitat ranges under both current and future climate scenarios (SSP1-2.6 and SSP5-8.5) for 2070 and 2090 in China. We aimed to address three key questions: (1) What are the current habitat ranges of these four *Symplocos* species in China? (2) What are the main drivers influencing their habitat suitability? (3) How will their habitat ranges change under future climate scenarios? We hypothesized that the current habitat distributions of these species are likely similar, concentrated in subtropical areas, but will vary significantly in the future due to species-specific responses to climate change. Furthermore, we expected both climatic and anthropogenic factors would significantly shape these species’ habitat range shifts. Our findings would provide critical insights for biodiversity conservation and prioritizing germplasm collection in China, offering a framework for addressing climate-driven challenges in forest ecosystems globally.

## 2. Materials and Methods

### 2.1. Species Distribution Data Pre-Processing

**Figure 1:**
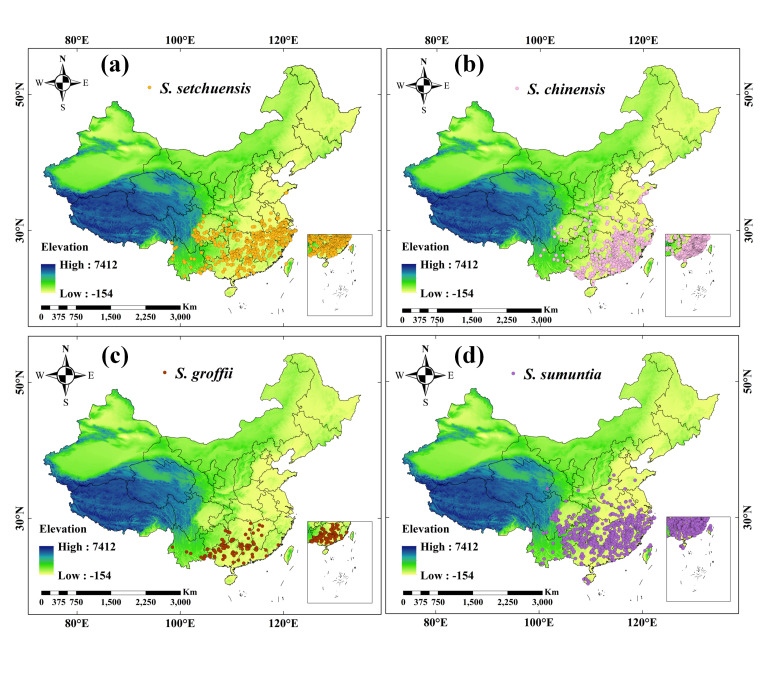
Geographical distribution of four *Symplocos* species in China, along with the regional zoning map. (**a**) Distribution of *S. setchuensis* (yellow); (**b**) Distribution of *S. chinensis* (pink); (**c**) Distribution of *S. groffii* (red); (**d**) Distribution of *S. sumuntia* (purple).

To obtain the occurrence records for *S. setchuensis*, *S. chinensis*, *S. groffii*, and *S. sumuntia* in China, we integrated specimen data from three open-source databases, including the Chinese Virtual Herbarium (CVH, [49]), the Global Biodiversity Information Facility (GBIF, [50]), and the Specimen Resources Sharing Platform for Education. We collected a total of 2408 specimen records for four *Symplocos* species, with specific tallies for each: *S. setchuensis* (650 records), *S. chinensis* (633 records), *S. groffii* (248 records), and *S. sumuntia* (877 records) from 1950 to 2024. For records without geographic coordinates but with accurate place names, we employed the map location website for location queries [51]. Moreover, duplicate and ambiguous geographical information records were manually removed after verification. To mitigate spatial autocorrelation from dense occurrence records, we applied a sparse processing technique using ArcGIS 10.8, ensuring each 5 km × 5 km grid cell retained only one point. Ultimately, we obtained a total of 520 occurrence records for *S. setchuensis*, 539 for *S. chinensis*, 106 for *S. groffii*, and 757 for *S. sumuntia* across China (Figure 1). To better illustrate the potential habitat ranges of the four *Symplocos* species, we categorized all provinces in China into six subregions: northeastern (NE), northwestern (NW), northern (N), eastern (E), southwestern (SW), and central southern (CS) China.

### 2.2. Environmental and Anthropogenic Parameters

To model the current and future habitat ranges of *S. setchuensis*, *S. chinensis*, *S. groffii*, and *S. sumuntia*, we integrated a total of 30 key predictors, including 19 bioclimatic variables, 3 topographic variables, 6 soil variables, and 2 anthropogenic factors (see Table S1 for details). First, the bioclimate data utilized encompass current (1970–2000) and future climate scenarios (2070s: average for 2061–2080; 2090s: average for 2081–2100) from the Worldclim dataset [52]. We used future climate projections based on eight global climate models (GCMs) from the Coupled Model Intercomparison Project Phase 6 (CMIP6, [53]) to predict the future habitat ranges in China of the four species, including ACCESS-CM2 [54], BCC-CSM2-MR [55], CMCC-ESM2 [56], GISS-E2-1-G [57], IPSL-CM6A-LR [58], MIROC6 [59], MPI-ESM1-2-HR [60], and MRI-ESM2-0 [61]. To mitigate the uncertainties of climate change, future bioclimatic data were collated and averaged across the aforementioned GCMs. Furthermore, to accurately predict the future habitat ranges of the four species, we specifically selected two distinct shared socioeconomic pathways (SSPs) climate scenarios of GCMs. SSP1–2.6 represents a sustainable development pathway with low greenhouse gas emissions, where atmospheric CO_2_ concentrations are projected to stabilize approximately 440 ppm by 2100. In contrast, SSP5–8.5 depicts a fossil fuel–intensive development trajectory with high emissions, leading to CO_2_ concentrations exceeding 1000 ppm by 2100 [62].

In addition, the topographic variables, including elevation, aspect, and slope, were obtained from the Geospatial data cloud with a spatial resolution of 2.5 min [63]. Meanwhile, we selected six soil crucial variables for plant growth, such as soil pH and total nitrogen, from the SoilGrids database with a sampling depth of 5 to 15 cm [64]. To account for anthropogenic influences, we included the human footprint index (HFI, 1993–2009) and the human build index (HBI, 1990–2017) in our model [17,65]. The HFI comprehensively measures the human influence on the global surface, encompassing the effects of built environments, population density, electric infrastructure, cropland, pasture lands, roads, railways, and navigable waterways [49]. The HBI, in contrast, quantifies the degree of human modification of terrestrial ecosystems based on long-term land use dynamics and infrastructure expansion. These two indices were selected because they jointly capture both the intensity and spatial extent of human disturbances, offer consistent temporal coverage at the global scale, and minimize data redundancy. All predictors were used at a spatial resolution of 5 km × 5 km.

To mitigate multicollinearity among predictor variables and enhance the accuracy of the SDMs [66], we employed the ‘ggcorr’ function from the corrplot R package and the ‘vifstep’ function from the usdm R package. Variables exhibiting a Spearman correlation coefficient (>0.8) and variance inflation factor (VIF > 10) were removed. As a result, we retained 12, 10, 13, and 13 key predictors for *S. setchuensis*, *S. chinensis*, *S. groffii*, and *S. sumuntia*, respectively (Table S3).

### 2.3. Model Optimization and Evaluation

The maximum entropy (MaxEnt) algorithm was employed to project the current and future habitat ranges for *S. setchuensis*, *S. chinensis*, *S. groffii*, and *S. sumuntia* [67]. The number of background points was determined based on the occurrence records of each species, with 2000 background points used for the less sampled *S. groffii* (106 occurrence records), and 3000 background points for the more sampled *S. setchuensis* (520 occurrence records), *S. chinensis* (539 occurrence records), and *S. sumuntia* (757 occurrence records). To minimize sampling bias and ensure robust model evaluation, we randomly partitioned the occurrence and background data into a training set (80%) and a testing set (20%) for MaxEnt modeling. Model accuracy was optimized by tuning the feature class (FC) combinations and regularization multiplier (RM) settings, as default parameters may lead to overfitting [46,68,69]. We used the ENMeval package in R to evaluate model performance, generating 30 candidate models by exploring six RM values (0.5–3.0, interval 0.5) and five FC combinations (LQ, LH, LP, LQP, and LQPH, where L = linear, Q = quadratic, H = hinge, and P = product) [70,71]. To validate model robustness, we further applied 10 repetitions of a 5-fold cross-validation method to assess prediction stability across species.

The optimal model was evaluated based on two evaluation metrics: the area under the receiver operating characteristic (ROC) curve (AUC) and true skill statistics (TSS) [72,73]. The AUC value is a robust, threshold-independent measure, ranging from 0 to 1, with higher values indicating better model performance [74]. We classified AUC values as follows: AUC ≤ 0.60 as fail, 0.60 < AUC ≤ 0.70 as poor, 0.70 < AUC ≤ 0.80 as fair, 0.80 < AUC ≤ 0.90 as good, and 0.90 < AUC ≤ 1.0 as excellent [75]. Meanwhile, TSS was also used as an additional measure of model performance, calculated as the true positive rate minus the false positive rate, with values between 0.4 and 0.6 indicating fair performance and values from 0.6 to 1 indicating good performance [76,77]. The Kappa coefficient was incorporated to quantify the agreement between predicted and observed occurrences beyond random chance, thereby offering a more balanced view of model accuracy when presence–absence ratios are unequal. In addition, the OR10 metric evaluates the proportion of observed presences omitted at a 10% training threshold, which serves as an important indicator of model overfitting and omission errors. Lower OR10 values indicate that the model effectively captures most known occurrences without excessive generalization.

To evaluate the relative importance of environmental and anthropogenic predictors on the potential habitat ranges of the four *Symplocos* species, we utilized the Jackknife test to assess the importance of each predictor variable in the optimal model [67]. Furthermore, we constructed response curves to explore the relationship between key variables and the habitat suitability of the four *Symplocos* species.

### 2.4. Classification of Suitable Habitat

For future habitat suitability predictions of four *Symplocos* species under SSP1-2.6 and SSP5-8.5 scenarios, climatic variables (e.g., temperature, precipitation) were varied, while topographic (e.g., elevation, slope), soil (e.g., moisture, pH), and human impact (e.g., human footprint index) variables were held constant, as these are assumed to remain relatively stable over the short term. To determine the trend of four *Symplocos* habitat suitability over time, we created binary habitat suitability maps for the current and future periods using the “ENMeval” package in R v4.2.1. To quantify the potential habitat probability of *S. setchuensis*, *S. chinensis*, *S. groffii*, and *S. sumuntia*, we assigned values ranging from 0 to 1 and then reclassified these values into four categories in ArcGIS 10.8 [78]. Habitat suitability was quantified using the SDM Toolbox in ArcGIS 10.8 and subsequently classified into four discrete categories: unsuitable (0–0.2), low suitability (0.2–0.4), medium suitability (0.4–0.6), and high suitability (>0.6) [79,80]. To maintain consistency and facilitate cross-species comparisons, a uniform threshold scheme was applied across all models. Additionally, we compared the differences in habitat ranges for the four species between the current period and future climatic scenarios, specifically under two SSP scenarios (SSP1-2.6 and SSP5-8.5) for the years 2070s and 2090s. To evaluate habitat changes in the four species under these future climatic scenarios, we classified the results into three trends: contraction, unchanged, and expansion.

## 3. Results

### 3.1. The Optimal Model and Its Accuracy

Our study determined the best Maxent model parameter combinations for the four *Symplocos* species in China (Table 1). Specifically, the optimal Maxent model for *S. setchuensis*, *S. chinensis*, *S. groffii* were characterized by a feature combination (FC) of LH and a regularization multiplier (RM) of 3.0, while *S. groffii* employed the LQPH feature combination with an RM of 3 (Table 1). The models demonstrated excellent performance, with test AUC values ranging from 0.93 to 0.95, test TSS values between 0.78 and 0.86, Kappa values from 0.45 to 0.62, and 10% omission rates (OR10) between 0.11 and 0.19 (Table 1). These metrics collectively indicate robust predictive accuracy for the suitable habitats of *S. setchuensis*, *S. chinensis*, *S. groffii*, and *S. sumuntia*, supporting their reliability for conservation planning.

### 3.2. Current Suitable Habitat Distribution of the Four Symplocos Species

**Figure 2:**
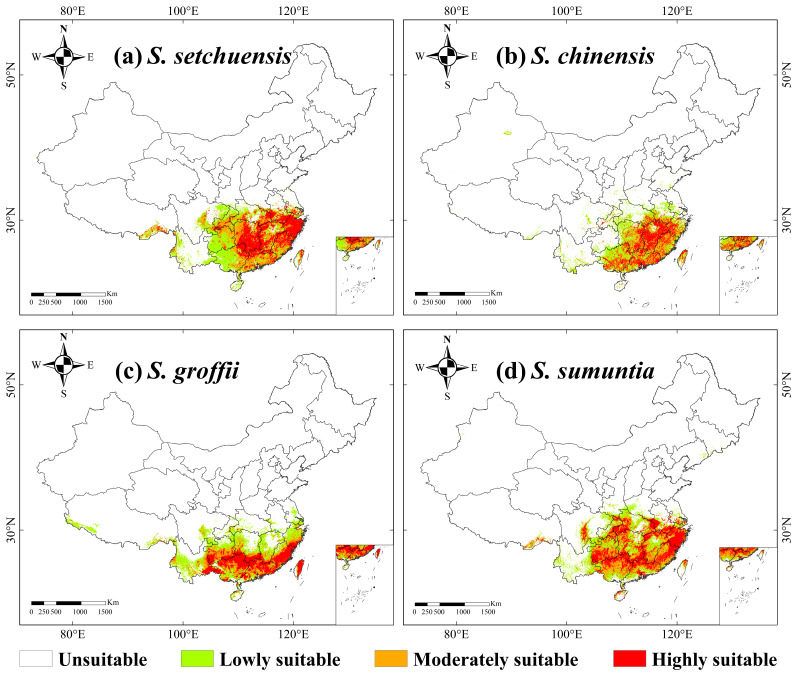
Current habitat ranges of four *Symplocos* species in China. The color scale indicates the probability of habitat suitability: red denotes highly suitable areas (probability > 0.6), orange denotes moderately suitable areas (probability 0.4–0.6), green indicates low suitability (probability 0.2–0.4), and white represents unsuitable areas. (**a**) *S. setchuensis*; (**b**) *S. chinensis*; (**c**) *S. groffii*; (**d**) *S. sumuntia*.

Our results revealed that the current habitat ranges of the four *Symplocos* species were primarily located in the subtropical and tropical regions of southern China, exhibiting notable differences in areas of high habitat suitability (Figure 2). For *S. setchuensis*, the total suitable habitat area was 15.35 × 10^5^ km^2^, accounting for 15.99% of the total land area of China (Table S2), with the highest suitability predominantly in central south and east China (Figure 1 and Figure 2a). This distribution aligns with the species’ preference for humid, mid-elevation montane forests, where precipitation and temperature gradients support its physiological requirements. Meanwhile, *S. chinensis* had a total suitable habitat area of 10.78 × 10^5^ km^2^ (11.23% of China), with the highly suitable areas concentrated in the southern part of central south and east China (Figure 1 and Figure 2b). Such patterns reflect adaptation to warmer, lowland subtropical climates with high rainfall, facilitating seed dispersal and growth. Moreover, the total suitable habitat area for *S. groffii* was 12.74 × 10^5^ km^2^ (13.27% of China), with the highest habitat suitability found primarily in South China, such as Zhejiang, Fujian, Guangdong, and Guangxi provinces (Figure 1 and Figure 2c). This concentration in coastal and southern provinces corresponds to the species’ reliance on maritime-influenced ecosystems, enhancing resilience to seasonal variability. Finally, the total suitable habitat area of *S. sumuntia* was 15.72 × 10^5^ km^2^ (16.38% of China), with the highly suitable ranges mainly distributed in southwestern, central southern, and eastern China (Figure 1 and Figure 2d). These broader ranges indicate tolerance to diverse topographic conditions, from karst landscapes to river valleys, promoting genetic diversity across fragmented habitats.

### 3.3. Important Variables Affecting the Habitat Ranges

**Figure 3:**
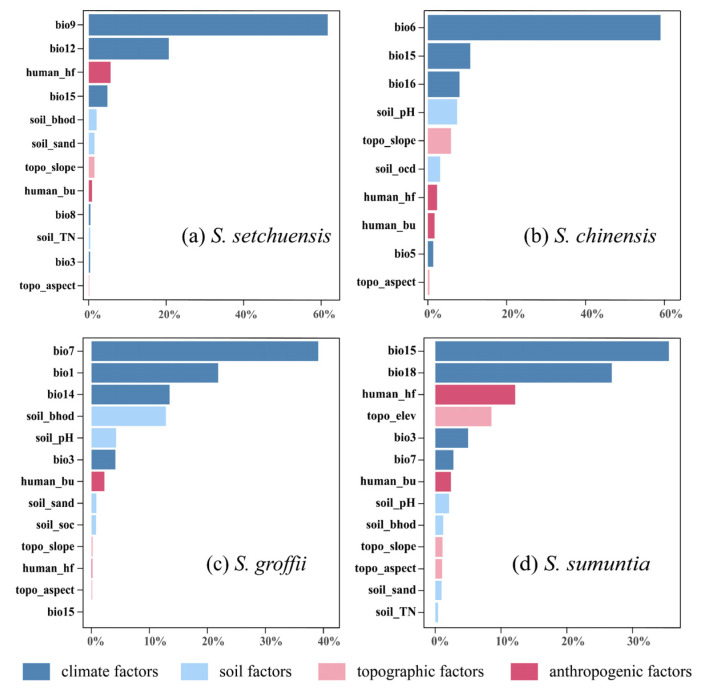
Permutation importance of environmental and anthropogenic variables used to predict the current habitat ranges of four *Symplocos* species. Colors represent variable categories: Dark blue represents climatic factors, sky blue represents soil variables, sky pink denotes topographical factors, and dark pink indicates anthropogenic variables. (**a**) *S. setchuensis*; (**b**) *S. chinensis*; (**c**) *S. groffii*; (**d**) *S. sumuntia*.

The habitat ranges of *S. setchuensis*, *S. chinensis*, *S. groffii*, and *S. sumuntia* were primarily influenced by climatic factors, followed by soil, anthropogenic, and topography factors (Figure 3). For *S. setchuensis*, the top four contributing variables were the mean temperature of the driest quarter (bio9, 61.7%), mean annual precipitation (bio12, 20.7%), human footprint (human_hf, 5.7%), and precipitation seasonality (bio15, 4.8%), collectively accounting for 92.9% of variation. The dominance of bio9 reflects this species’ sensitivity to temperature stress during dry periods, likely limiting its distribution to warmer, humid montane forests, while bio12 underscores its reliance on consistent water availability for growth. In addition, *S. chinensis* was most influenced by the minimum temperature of the coldest month (bio6, 59.0%), precipitation seasonality (bio15, 10.7%), precipitation of the wettest quarter (bio16, 8.0%), and soil pH (7.4%), explaining 85.2% of the variation. The prominence of bio6 indicates a strong dependence on frost-free conditions, typical of lowland subtropical habitats, with soil pH influencing nutrient availability for root development. For *S. groffii*, the key factors were the temperature annual range (bio7, 39.1%), mean annual temperature (bio1, 21.8%), and precipitation of the driest month (bio14, 13.5%), and soil bulk density of the fine earth fraction (soil bhod, 12.8%), accounting for 87.3% of variation. The high contribution of bio7 suggests adaptation to stable coastal climates with moderate temperature fluctuations, while soil bulk density likely affects rooting depth and water retention in southern provinces. Lastly, the habitat range of *S. sumuntia* was mainly influenced by precipitation seasonality (bio15, 35.5%), precipitation of the warmest quarter (bio18, 26.8%), human footprint (human_hf, 12.2%), and elevation (8.5%), which collectively accounted for 83.1% of the variation. The influence of bio15 and bio18 highlights this species’ adaptation to monsoon-driven climates, with elevation shaping its distribution across diverse topographic gradients, from karst to valley systems.

**Figure 4:**
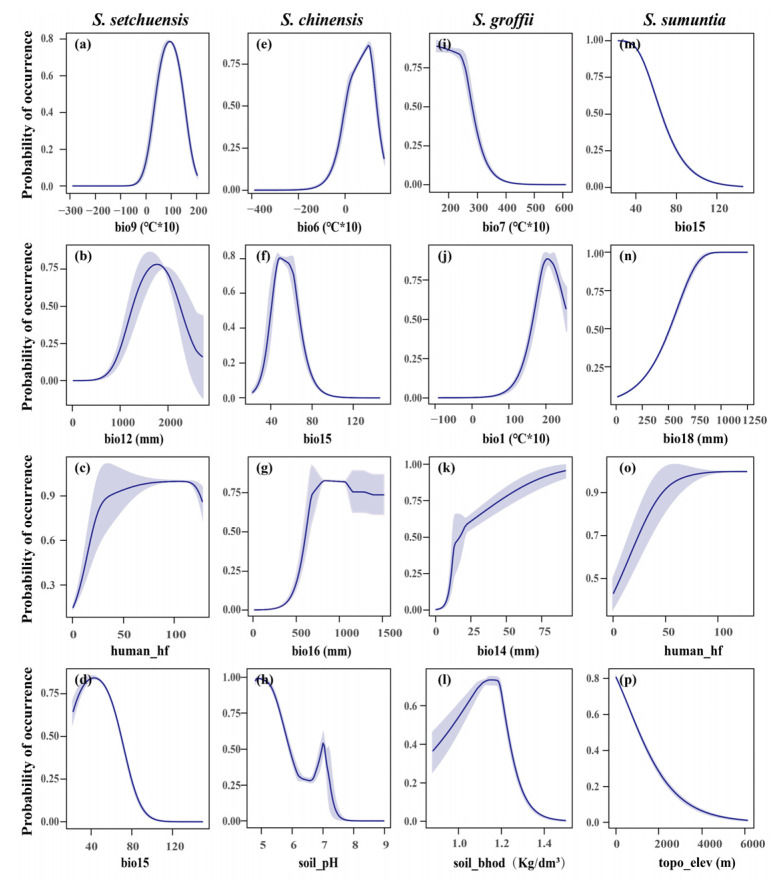
Response curves for the top four contributing predictors in the species distribution model of four *Symplocos* species under the current climate conditions in China. The curves illustrate the relationship between environmental variables and the probability of occurrence for each species, with confidence bands indicated by shaded regions. (**a**) Mean temperature of the driest quarter (bio9); (**b**) Annual precipitation (bio12); (**c**) human footprint (human_hf); (**d**) Precipitation seasonality (bio15); (**e**) Minimum temperature of the coldest month; (**f**) Precipitation seasonality (bio15); (**g**) Precipitation of the wettest quarter (bio16); (**h**) Soil pH; (**i**) Temperature annual range (bio7); (**j**) Annual mean temperature (bio1); (**k**) Precipitation of the driest month (bio14); (**l**) Soil bulk density (soil_bhod); (**m**) Precipitation seasonality (bio15); (**n**) Precipitation of the warmest quarter (bio18); (**o**) human footprint (human_hf); (**p**) Elevation (topo_elev).

Furthermore, the Jackknife test illustrated the impact of each crucial variable on the potential habitat range of the four *Symplocos* species (Figure 4). For *S. setchuensis*, habitat suitability showed a unimodal relationship with mean annual precipitation (bio12) and mean temperature of the driest quarter (bio9), peaking at intermediate values, while negatively correlated with precipitation seasonality (bio15) and positively with the human footprint index (human_hf, Figure 4a–d). These patterns suggest *S. setchuensis* thrives in stable, humid montane environments but is sensitive to irregular rainfall, with some tolerance for moderate anthropogenic disturbance. Similarly, for *S. chinensis*, unimodal relationships were found with the minimum temperature of the coldest month (bio6) and precipitation seasonality (bio15), while habitat suitability was negatively correlated with soil pH and positively with precipitation of the wettest quarter (bio16, Figure 4e–h). This reflects adaptation to frost-free subtropical lowlands, where high rainfall supports growth, but acidic soils may limit nutrient uptake. For *S. groffii*, habitat suitability positively correlated with precipitation of the driest month (bio14) and negatively with the temperature annual range (bio7), with unimodal relationships for mean annual temperature (bio1) and soil bulk density (Figure 4i–l). These trends indicate a preference for stable, moist coastal climates, with soil structure influencing root establishment. In addition, for *S. sumuntia*, habitat suitability positively correlated with precipitation of the warmest quarter (bio18) and the human footprint index (human_hf), while negatively correlated with bio15 and elevation (Figure 4m–p). This indicates that *S. sumuntia* thrives in monsoon-driven climates and tolerates low to moderate anthropogenic disturbances, such as selective logging or agricultural expansion, likely due to its adaptability to altered landscapes, but is sensitive to variable rainfall and high elevations.

### 3.4. Potential Suitable Habitat Ranges in the Future

**Figure 5:**
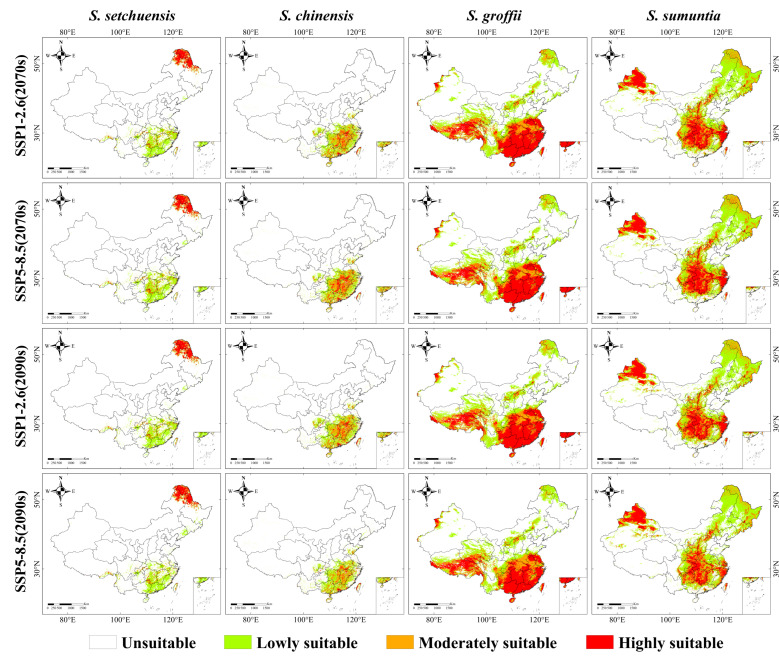
Potential habitat ranges of four *Symplocos* species under future climate scenarios in China. The color legend is as follows: green denotes lowly suitable, orange indicates moderately suitable, and red represents highly suitable.

**Figure 6:**
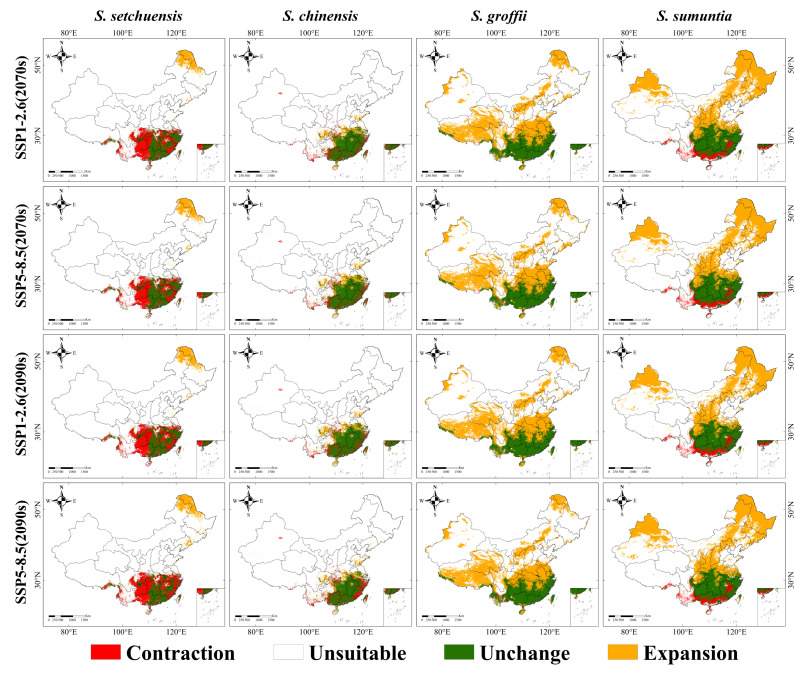
Potential habitat range changes in four *Symplocos* species under SSP1-2.6 (2070s), SSP5-8.5 (2070s), SSP1-2.6 (2090s), and SSP5-8.5 (2090s) scenarios, compared to their current habitat distributions in China. The color legend is as follows: dark green denotes unchanged areas, orange indicates expansion areas, red represents contraction areas, and white represents unsuitable areas.

We observed large differences in the responses of the habitat ranges of four *Symplocos* species to multiple future climate scenarios (SSP1-2.6 and SSP5-8.5 during the 2070s and 2090s, Figure 5, Figure 6 and Figure S1). For *S. setchuensis*, the total mean future suitable habitat area was projected to be 9.65 × 10^5^ km^2^, with highly suitable habitats concentrated in Heilongjiang and Inner Mongolia provinces, while low and moderately suitable habitats would remain in their current habitat ranges. This northward shift suggests a response to warming temperatures, potentially driven by increased winter temperatures in northeastern China. Conversely, the potential habitat range of *S. chinensis* was expected to remain within its current habitat range (mean future habitat area: 11.31 × 10^5^ km^2^), but the extent of highly suitable habitats would significantly decrease. This contraction of high-suitability areas indicates sensitivity to intensified climatic extremes, particularly in subtropical lowland regions. In addition, the total mean future suitable habitat area of *S. groffii* was anticipated to expand to 42.33 × 10^5^ km^2^, with a substantial increase in highly suitable habitats in southwest China. This expansion reflects the species’ adaptability to warmer, wetter conditions in montane regions, likely facilitated by increased precipitation under SSP5-8.5. Similarly, for *S. sumuntia*, the total mean future suitable habitat area was projected to be 39.58 × 10^5^ km^2^, with highly suitable habitats maintained in the current habitat areas (Figure 5, Figure 6 and Figure S2 and Table S2). The stability of its high-suitability areas, coupled with moderate expansion, suggests resilience to climate variability across diverse topographic landscapes. Collectively, *S. setchuensis* shows a northward shift, *S. chinensis* faces habitat contraction, while *S. groffii* and *S. sumuntia* expand, driven by species-specific responses to temperature and precipitation changes.

Further, our results demonstrated notable differences in the expansion and contraction trends of all four species under future climate scenarios (Figure 6 and Table S2). The habitat range of *S. setchuensis* was anticipated to contract by 5.57 × 10^5^ km^2^, with loss mainly in its current habitat range’s western and eastern regions. This contraction likely results from increased temperature stress in these regions, limiting suitability for this montane species. In contrast, *S. chinensis* was expected to maintain a relatively stable trend under future climate scenarios, with a small expansion of 0.56 × 10^5^ km^2^ in the northern and slight contraction in the east. This stability reflects its adaptation to subtropical climates, though constrained by intensified climatic extremes. *S. groffii*, on the other hand, was expected to exhibit a strong expansion trend, with its habitat increasing by 29.68 × 10^5^ km^2^, especially in central and northern China. This expansion is likely driven by *S. groffii*’s tolerance to cooler temperatures and its ability to exploit increased rainfall projected for northern China. This expansion is driven by its tolerance to cooler temperatures and increased rainfall, aligning with moist, temperate conditions in these regions. Similarly, *S. sumuntia* was forecasted to expand by 23.93 × 10^5^ km^2^ in central, northern, and northeastern China, with minimal southern contraction. This expansion reflects its adaptability to increased precipitation and moderate temperature rises, enabling it to thrive across varied topographic landscapes, including karst and valley systems.

## 4. Discussion

The *Symplocos* genus, diverse and widely distributed south of the Yangtze River, is vital for subtropical forest biodiversity and carbon sequestration in China [1,3,5]. Based on multi-source data and optimal Maxent models, this study quantified and compared the potential habitat ranges of the four *Symplocos* species in China, *S. setchuensis*, *S. chinensis*, *S. groffii*, and *S. sumuntia*, under the current and future climate scenarios. In addition, we further assessed the relative importance of human activities and environmental factors such as climate on the habitat suitability of the four *Symplocos* species.

### 4.1. Model Predictive Performance

Accurately predicting the potential habitat range for key plant species is crucial for biodiversity conservation and forest management [81,82,83]. Metrics such as AUC, TSS, Kappa, and omission rates (OR10) are widely recognized as robust indicators for assessing the predictive performance of species distribution models [81,84,85]. Our optimized MaxEnt models exhibited high AUC (0.93–0.95) and TSS (0.78–0.86) values, complemented by moderate-to-high Kappa (0.45–0.62) and low OR10 (0.11–0.19), suggesting strong predictive ability while maintaining low overfitting and bias risk [86]. The high AUC values indicate excellent discrimination between suitable and unsuitable habitats, a prerequisite for reliable mapping in complex subtropical ecosystems [67]. Similarly, the elevated TSS values confirm a strong balance between omission and commission errors, supporting accurate identification of core distribution zones [73]. Moderate-to-high Kappa values demonstrate substantial agreement beyond random expectation, reinforcing the reliability of habitat classification despite sampling variations [73]. Low OR10 values indicate minimal omission errors at the 10% training threshold, underscoring the models’ ability to capture species’ environmental niches without excessive overfitting, enhancing confidence in future projections for adaptive forest management [87]. Unlike default MaxEnt settings, our optimized models—using LH features for *S. setchuensis*, *S. chinensis*, and *S. sumuntia*, and LQPH for *S. groffii* (all with RM = 3.0)—substantially improved predictive reliability across all evaluation metrics [69,88,89]. These robust models enable confident forecasting of the current and future habitat ranges of the four *Symplocos* species, providing a valuable tool for prioritizing conservation efforts in regions vulnerable to climate change and supporting sustainable forest management.

### 4.2. Habitat Distribution and Key Predictors Under the Current Environment

Our results showed that the habitat ranges for the four *Symplocos* species in China are mainly distributed in subtropical regions, with highly suitable habitat areas concentrated in central south and east China. These findings are consistent with the current observed distribution of the *Symplocos* species, supporting the accuracy of our predictions [90,91,92]. Further, we identified that climate factors are more influential in shaping the habitat distribution of *S. setchuensis*, *S. chinensis*, *S. groffii*, and *S. sumuntia* than human, topographic, and soil drivers, which aligns with recent studies [93,94,95].

Distinct climatic sensitivities were observed among the four *Symplocos* species. For instance, the habitat range of *S. setchuensis* was most influenced by the mean temperature of the driest quarter (bio9), while the habitat distribution of *S. chinensis* was shaped by the minimum temperature of the coldest month (bio6), following a single-peak pattern. These findings suggest that both excessively low and high temperatures are detrimental to plant survival and regeneration, causing frost damage and disrupting seed dormancy, respectively [96,97]. Meanwhile, the habitat suitability of *S. groffii* showed a decline with increasing temperature annual range (bio7), likely due to the stress of extreme temperatures on critical physiological processes such as seed germination and photosynthesis [98,99]. Furthermore, precipitation was also identified as a key determinant of species range shifts [97,100]. Our results revealed that precipitation seasonality (bio15), the mean annual precipitation (bio12), precipitation of the driest month (bio14), precipitation of the wettest quarter (bio16), and precipitation of the warmest quarter (bio18) had a strong influence on the habitat distribution of the four *Symplocos* species, particularly impacting *S. sumuntia*. Specifically, bio14, bio16, and bio18 positively affected the habitat distribution of *Symplocos* species, aligning with recent studies [100,101]. Notably, the relationship between bio12 and *S. setchuensis* habitat suitability is found to be unimodal, suggesting that excessive rainfall can lead to anaerobic soil conditions that impede root metabolic processes, thus limiting plant growth and seedling survival [102,103]. In contrast, we observed that bio15 had a negative effect on both *S. sumuntia* and *S. setchuensis*, implying that stable precipitation is essential for the survival and dispersal of these species, while extreme weather events may exert a disproportionately strong influence on *Symplocos* species [104,105].

In addition to climate factors, our findings demonstrated that topographic factors (e.g., elevation) and soil factors (e.g., soil pH and soil bulk density) had a significant effect on the habitat range of *Symplocos* species, aligning with recent studies [106,107]. Specifically, a negative correlation between elevation and the habitat suitability of *S. sumuntia* was observed. A recent study has shown that leaf morphological characteristics of *Symplocos* species are strongly influenced by abiotic conditions, with a decrease in leaf size observed at higher elevations [108]. This indicates that increased elevation may impede the photosynthetic intensity of *S. sumuntia*, reducing its habitat suitability. Moreover, the habitat suitabilities of *S. chinensis* and *S. groffii* were significantly modulated by soil pH and soil bulk density, respectively. The habitat suitability of *S. chinensis* consistently declined with increasing soil pH, likely because elevated soil pH restricts the bioavailability of essential micronutrients such as Manganese, Copper, and Zinc, thereby impeding plant growth [109]. The unimodal relationship between soil bulk density and the fitness of *S. groffii* can be attributed to the fact that excessively high soil bulk density reduces soil porosity, obstructing gas exchange and water infiltration. This impairs root respiration and water absorption [110,111]. Conversely, excessively low soil bulk density hinders the plant’s ability to establish roots effectively. These findings are supported by previous studies showing that soil characteristics significantly affect plant distribution [112,113,114].

The habitat ranges of species are often shaped by human activities, which can drive both declines and expansions [15,115]. Our results revealed a positive correlation between human footprints and the habitat suitability of *S. setchuensis* and *S. sumuntia*. This suggests that effective human interventions, such as afforestation, strategic forest management, and habitat restoration measures, may play a pivotal role in maintaining or even expanding the range and abundance of these species [116,117,118]. On the other hand, this pattern may be explained by the increased adaptability of these species to environmental changes driven by human activities [119,120]. In line with our findings, a recent study has also demonstrated that human activities may promote plant diversity and range expansion by enhancing colonization and seed dispersal [121].

### 4.3. Changes in the Distribution Habitats of Symplocos Species Under Future Climatic Scenarios

Our findings highlight distinct responses in the potential habitat ranges of the four *Symplocos* species under SSP1-2.6 and SSP5-8.5 scenarios for the 2070s and 2090s, reflecting broader biogeographic patterns driven by species-specific adaptations to climate change [122,123]. *S. setchuensis* is projected to experience significant habitat contraction, particularly under SSP5-8.5 in the 2090s, with losses in its current montane ranges and limited northward expansion into cooler northeastern regions. This poleward shift aligns with global trends of montane species tracking cooler thermal niches, potentially intensifying competition and altering community dynamics in northern ecosystems [124,125,126]. Conversely, *S. chinensis* is expected to maintain relative habitat stability with slight northward expansion, though its highly suitable areas may decline under SSP5-8.5 in the 2090s. We hypothesize that this stability stems from its adaptation to humid subtropical environments, which may buffer against temperature increases, though this requires further validation due to limited direct evidence [127,128].

Contrary to predictions of widespread habitat loss under climate warming [129,130,131], *S. groffii* and *S. sumuntia* are projected to undergo significant habitat expansion, driven by their adaptability to increased precipitation, moderate temperature rises, and diverse topographic conditions [32,132,133]. Expansion of *S. groffii* into northeast and northwest China reflects tolerance for wetter, temperate climates and ability to exploit complex terrains like karst landscapes, potentially enhancing regional biodiversity by colonizing new ecological niches [134]. Similarly, northward expansion of *S. sumuntia*, particularly into northeastern China, is facilitated by resilience to monsoon-driven precipitation and topographic heterogeneity, though highly suitable habitats are constrained under SSP5-8.5 due to intensified drought stress [135,136]. These expansions highlight a broader biogeographic trend where species with flexible ecological tolerances act as pioneers in newly suitable regions, influencing ecosystem structure and function [91].

Overall, our results indicate marked differences in the direction and magnitude of habitat range shifts among *Symplocos* species under future climate change, which is consistent with prior findings [137,138]. These differences are likely due to species-specific physiological characteristics and environmental adaptations [90,139,140]. Further, species habitat distribution is not solely driven by temperature but rather by a complex interaction of ecological factors [141,142], where the combined effects of temperature and moisture play a key role in determining habitat suitability [100]. SSP5-8.5 represents a climate scenario of rapid global economic expansion driven by fossil fuel extraction and energy-intensive lifestyles [143,144]. Our findings reveal that under the SSP5-8.5 climate scenario, the habitat ranges of the four *Symplocos* species face varying degrees of restriction, implying that high greenhouse gas emissions will weaken their growth and survival, thereby limiting them to narrower suitable habitat ranges [145,146].

### 4.4. Limitations and Prospects

Our study provides a framework for predicting and comparing the current and future habitat ranges of the four *Symplocos* species, yet some limitations persist. First, the occurrence data used in our models may be subject to sampling bias, as records are often concentrated in more accessible or frequently surveyed areas, potentially leading to underrepresentation of remote habitats and skewed suitability predictions [147,148]. Additionally, resolution mismatches between predictors—such as coarse-scale bioclimatic variables and finer-scale soil or topographic data—could introduce uncertainties in habitat modeling, affecting the precision of projections at local scales [79]. Second, species habitat distributions are influenced not only by climate, soil, topography, and anthropogenic factors but also by other factors such as biological interaction and dispersal limitations [149]. Due to the challenge of quantifying some of these variables and the risk of multicollinearity, our model only focuses on key drivers, which may result in deviations from the actual distribution patterns of the four *Symplocos* species. Third, potential habitat projections often exceed actual species distributions [150], potentially leading to an overestimation of the suitable habitat areas. To address these limitations, future studies should integrate higher-resolution data, broader species coverage, and biotic factors to enhance model accuracy.

Given the ecological and human utility of *Symplocos* species, additional human influences, such as their incorporation into managed plantings, warrant exploration. The projected northward expansion of *S. groffii* and *S. sumuntia* under future climate scenarios aligns with China’s afforestation initiatives, such as the Grain for Green Project. Strategic plantings of these species in central and northern China could facilitate range shifts, leveraging their adaptability to cooler temperatures and increased precipitation to enhance biodiversity in restored forests. For *S. setchuensis*, facing habitat contraction, urgent conservation actions include germplasm banking to preserve genetic diversity and protecting mesic montane habitats in central China. *S. chinensis*, with stable ranges, would benefit from habitat monitoring and invasive species control. These strategies, grounded in our ecological indicators, provide actionable pathways for integrating *Symplocos* into conservation and afforestation programs. Future studies should assess the feasibility and socio-economic impacts of human-assisted range shifts, ensuring robust management frameworks for sustaining *Symplocos* diversity under global change.

## 5. Conclusions

This study provides novel insights into the habitat dynamics of four representative Symplocos species in China, integrating climate change projections and human activity influences through species distribution modeling. We reveal clear geographical differentiation among species, reflecting their distinct ecological niches and climatic sensitivities. Currently, all species are predominantly distributed in the subtropical regions of southern China, with varying extents of suitable habitat. Moreover, our findings underscore that climatic extremes, particularly low-temperature tolerance and precipitation seasonality, are key determinants of species distribution, while human activities can exert both facilitative and constraining effects depending on species-specific adaptability. Under future climate scenarios, the four *Symplocos* species exhibited divergent range dynamics, with *S. groffii* and *S. sumuntia* projected to expand northwards, whereas *S. setchuensis* is likely to contract and shift towards central regions. These contrasting responses suggest that thermal adaptability and topographic buffering jointly mediate climate resilience within the genus. This study offers valuable insights into the combined effects of climate change and human activity on the habitat ranges of *Symplocos* species, informing strategies for prioritizing germplasm collection, forest conservation, and management in response to future climate changes.

## Figures and Tables

**Table 1:** Evaluation metrics of MaxEnt generated by ENMeval for four *Symplocos* species in China.

Species	FC	RM	AUC	TSS	Kappa	OR10
*S. setchuensis*	LH	3.00	0.94	0.80	0.58	0.13
*S. chinensis*	LH	3.00	0.94	0.78	0.58	0.12
*S. groffii*	LQPH	3.00	0.95	0.86	0.45	0.19
*S. sumuntia*	LH	3.00	0.93	0.78	0.62	0.11

## Data Availability

All the data have been archived in Figshare Digital Repository: https://doi.org/10.6084/m9.figshare.27683187.v1.

## Supplementary Materials

The following supporting information can be downloaded at https://www.mdpi.com/article/10.3390/plants14203200/s1.
