# Supplementary material for: Unraveling the Effects of Climate Change and Human Activity on Potential Habitat Range Shifts in Four Symplocos Species in China
Source: Plants (Basel). 2025 Oct 18;14(20):3200. doi: 10.3390/plants14203200 (PMC12567360; doi:10.3390/plants14203200)
Supplement: Supplementary file 1 [file plants-14-03200-s001.zip › plants-3859136-supplementary.pdf]

Supplementary information

Unraveling the effects of climate change and human activity on potential habitat range shifts of four *Symplocos* species in China

Eq. S1

This file includes:

Number of pages: 6; Number of tables: 3; Number of figures: 2;

Table S1. Environmental and anthropogenic factors for modeling the potential habitat ranges of four *Symplocos* species in China.

Code of factor	Description for factor	Unit
bio1	Annual mean temperature	℃*10
bio2	Mean diurnal range	℃*10
bio3	Isothermality ((bio2/bio7) *100)	-
bio4	Temperature seasonality (standard deviation*100)	-
bio5	Max temperature of the warmest month	℃*10
bio6	Min temperature of the coldest month	℃*10
bio7	Temperature annual range	℃*10
bio8	Mean temperature of the wettest quarter	℃*10
bio9	Mean temperature of the driest quarter	℃*10
bio10	Mean temperature of the warmest quarter	℃*10
bio11	Mean temperature of the coldest quarter	℃*10
bio12	Annual precipitation	mm
bio13	Precipitation of the wettest month	mm
bio14	Precipitation of the driest month	mm
bio15	Precipitation seasonality (coefficient of variation)	-
bio16	Precipitation of the wettest quarter	mm
bio17	Precipitation of the driest quarter	mm
bio18	Precipitation of the warmest quarter	mm
bio19	Precipitation of the coldest quarter	mm
topo_elev	Elevation	m
topo_aspect	Aspect	-
topo_slope	Slope	-
soil_bhod	Soil bulk density of the fine earth fraction	$kg{\cdot}dm^{-3}$
soil_soc	Soil organic carbon	$g{\cdot}kg^{-1}$
soil_ocd	Soil organic carbon density	$kg{\cdot}m^{-3}$
soil_sand	Soil sand fraction	%
soil_TN	Soil total nitrogen	$g{\cdot}kg^{-1}$
soil_pH	Soil pH	-
human_bu	Human build	-
human_hf	human footprint	-

Table S2 Parameter combinations with the highest AUC for the optimal Maxent model of four *Symplocos* species in China.

species	FC	RM	train_AUC	test_AUC	train_TSS	test_TSS
S. setchuensis	LQ	3.00	0.95	0.95	0.79	0.80
S. chinensis	LH	3.00	0.97	0.97	0.80	0.80
S. groffii	LH	3.00	0.97	0.97	0.84	0.85
S. sumuntia	LH	3.00	0.95	0.94	0.80	0.80

Note: AUC (area under the receiver operating characteristic curve) and TSS (true skill statistic) are metrics used to assess model accuracy, with higher values indicating better performance. FC represents the feature combination, and RM denotes the regularization multiplier.

Table S3. Predicted habitat suitable areas and dynamics for four *Symplocos* species under current and future climatic scenarios.

Species	Scenarios	Suitable areas /×10^5 km^2						Area change		
		MTSH	TSH	UH	LSH	MSH	HSH	Contracted	Increased	Unchanged
S. setchuensis	current	-	15.35	80.56	5.67	4.21	5.47	-	-	-
	2070s(ssp1-2.6)	9.65	9.56	86.36	6.10	1.24	2.22	8.28	2.61	7.09
	2070s(ssp5-8.5)		10.17	85.75	6.37	1.52	2.28	7.78	2.73	7.59
	2090s(ssp1-2.6)		9.48	86.43	6.07	1.21	2.20	8.41	2.66	6.96
	2090s(ssp5-8.5)		9.40	86.52	5.92	1.30	2.18	8.79	2.95	6.58
S. chinensis	current	-	10.78	85.14	4.15	3.06	3.57	-	-	-
	2070s(ssp1-2.6)	11.31	11.39	84.53	6.42	3.13	1.84	1.95	2.58	8.92
	2070s(ssp5-8.5)		11.69	84.23	5.86	3.46	2.38	1.86	2.80	9.01
	2090s(ssp1-2.6)		11.78	84.14	6.54	3.30	1.94	1.87	2.88	9.01
	2090s(ssp5-8.5)		10.39	85.53	6.39	2.63	1.37	2.61	2.25	8.25
S. groffii	current	-	12.74	83.18	5.52	2.98	4.24	-	-	-
	2070s(ssp1-2.6)	42.33	42.80	53.11	15.00	9.39	18.41	0.08	30.22	12.71
	2070s(ssp5-8.5)		42.47	53.44	15.03	9.03	18.41	0.18	30.00	12.60
	2090s(ssp1-2.6)		42.75	53.16	14.88	9.39	18.48	0.09	30.19	12.69
	2090s(ssp5-8.5)		41.31	54.60	14.39	9.79	17.13	0.27	28.92	12.50
S. sumuntia	current	-	15.72	80.19	5.09	4.72	5.91	-	-	-
	2070s(ssp1-2.6)	39.58	39.76	56.15	18.69	8.99	12.08	3.24	27.35	12.53
	2070s(ssp5-8.5)		40.02	55.89	18.32	8.96	12.75	3.26	27.63	12.51
	2090s(ssp1-2.6)		38.83	57.08	18.09	8.92	11.82	3.26	26.44	12.52
	2090s(ssp5-8.5)		39.69	56.22	19.80	9.28	10.61	3.30	27.34	12.48

Note: MTSH, the average total suitable area under various future climate scenarios; TSH, the total suitable habitat area under various climate scenarios; UH, unsuitable habitat area under various climate scenarios; LSH, lowly suitable habitat area under various climate scenarios; MSH, moderately suitable habitat area under various climate scenarios; HSH, highly suitable habitat area under various climate scenarios.

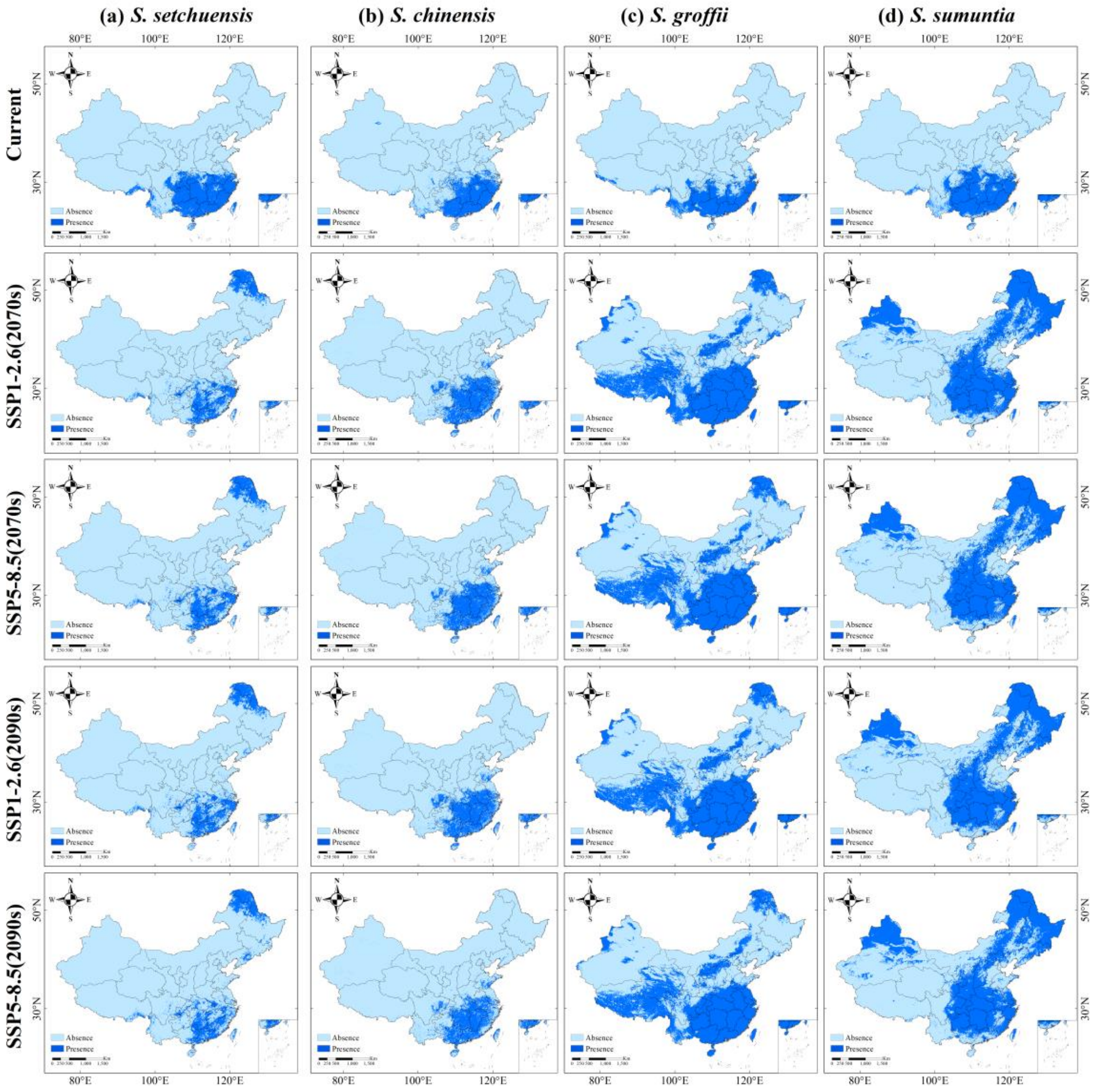

Fig. S1. Habitat distributions of *S. setchuensis* (a), *S. chinensis* (b), *S. groffii* (c), and *S. sumuntia* (d) under current and future climatic conditions in China. The future climate conditions include SSP1-2.6 (2070s), SSP5-8.5 (2070s), SSP1-2.6 (2090s), and SSP5-8.5 (2090s) scenarios. Light blue represents absence habitat areas, while dark blue represents presence habitat areas.

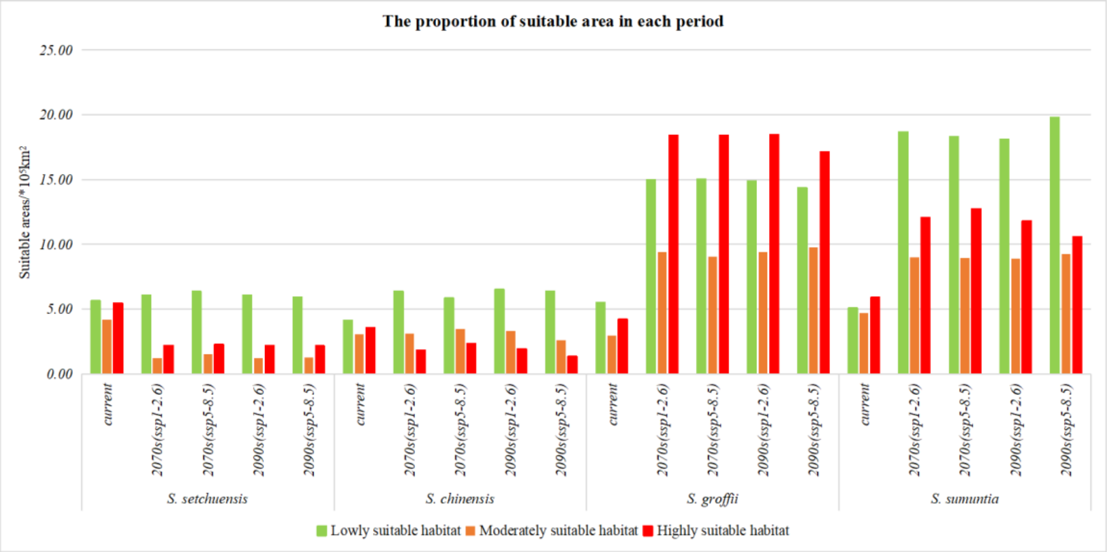

Fig. S2. Comparison of potentially habitat suitable areas for *S. setchuensis*, *S. chinensis*, *S. groffii*, and *S. sumuntia* in China under current and four future climate scenarios. The color scale indicates habitat suitability: red indicates highly suitable areas (probability > 0.6), orange represents moderately suitable areas (probability 0.4–0.6), and green represents lowly suitable areas (probability 0.2–0.4). SSP stands for shared socioeconomic pathways.
